# Role of Astrocytic Dysfunction in the Pathogenesis of Parkinson's Disease Animal Models from a Molecular Signaling Perspective

**DOI:** 10.1155/2020/1859431

**Published:** 2020-02-07

**Authors:** Lucas Udovin, Cecilia Quarracino, María I. Herrera, Francisco Capani, Matilde Otero-Losada, Santiago Perez-Lloret

**Affiliations:** ^1^Institute of Cardiological Research, University of Buenos Aires, National Research Council (ININCA-UBA-CONICET), Marcelo T. de Alvear 2270, C1122 Buenos Aires, Argentina; ^2^Pontifical Catholic University of Argentina, Buenos Aires, Argentina; ^3^Instituto de Ciencias Biomédicas, Facultad de Ciencias de la Salud, Universidad Autónoma de Chile, Chile; ^4^Department of Physiology, School of Medicine, University of Buenos Aires (UBA), Buenos Aires, Argentina

## Abstract

Despite the fact that astrocytes are the most abundant glial cells, critical for brain function, few studies have dealt with their possible role in neurodegenerative diseases like Parkinson's disease (PD). This article explores relevant evidence on the involvement of astrocytes in experimental PD neurodegeneration from a molecular signaling perspective. For a long time, astrocytic proliferation was merely considered a byproduct of neuroinflammation, but by the time being, it is clear that astrocytic dysfunction plays a far more important role in PD pathophysiology. Indeed, ongoing experimental evidence suggests the importance of astrocytes and dopaminergic neurons' cross-linking signaling pathways. The Wnt-1 (wingless-type MMTV integration site family, member 1) pathway regulates several processes including neuron survival, synapse plasticity, and neurogenesis. In PD animal models, Frizzled (Fzd) neuronal receptors' activation by the Wnt-1 normally released by astrocytes following injuries leads to *β*-catenin-dependent gene expression, favoring neuron survival and viability. The transient receptor potential vanilloid 1 (TRPV1) capsaicin receptor also participates in experimental PD genesis. Activation of astrocyte TRPV1 receptors by noxious stimuli results in reduced inflammatory response and increased ciliary neurotrophic factor (CNTF) synthesis, which enhances neuronal survival and differentiation. Another major pathway involves I*κ*B kinase (IKK) downregulation by ARL6ip5 (ADP-ribosylation-like factor 6 interacting protein 5, encoded by the cell differentiation-associated, JWA, gene). Typically, IKK releases the proinflammatory NF-*κ*B (nuclear factor kappa-light-chain-enhancer of activated B cells) molecule from its inhibitor. Therefore, by downregulating NF-*κ*B inhibitor, ARL6ip5 promotes an anti-inflammatory response. The evidence provided by neurotoxin-induced PD animal models guarantees further research on the neuroprotective potential of normalizing astrocyte function in PD.

## 1. Introduction

Parkinson's disease (PD) is the second most common neurodegenerative disease following Alzheimer's disease. It is characterized by loss of dopaminergic neurons in the midbrain [1, 2] and bradykinesia, rigidity, and tremor as main clinical symptoms. Regularly, patients also display nonmotor symptoms like cognitive impairment, mood disorders, sleep alterations, dysautonomia, and hallucinations [1].

Typical, though not only, histopathological changes are the progressive loss of the nigrostriatal dopaminergic pathway and hence of the striatal dopaminergic tone [2]. Over the last 40 years, administration of the amino acid precursor of dopamine L-DOPA (L-3,4-dihydroxy-L-phenylalanine) to parkinsonian patients has been considered the most effective symptomatic treatment [3].

Abnormal accumulation of misfolded protein aggregates [4] as the Lewy bodies, made of *α*-synuclein [5], appears to be one of the physiopathological hallmarks of the disease. One major target of *α*-synuclein is Rab-1 (a member of the Ras superfamily of monomeric G proteins, Rab GTPase family), a key molecular switch of the endoplasmic reticulum-Golgi traffic pathway [6]. The *α*-synuclein accumulation-induced endoplasmic reticulum stress is likely a leading disruptive mechanism, responsible for the so-called “unfolded protein response” adaptive reaction [7], cytoprotective when moderate but deleterious when highly activated [8, 9]. Accumulation of *α*-synuclein may also originate abnormal synaptic connectivity or synaptopathy at nigrostriatal pathways and intrastriatal interneuronal connections, presumably most apparent at the initial stages of the disease.

Notwithstanding the fact that astrocytes are the most abundant glial subtype and are critical for brain function, only a few studies have historically focused on their putative role in neurodegenerative diseases like PD. Recently, however, several studies have reported that genes known to have a causative role in PD are expressed in astrocytes and have important roles in their function [10], suggesting that astrocyte dysfunction may be relevant for PD development. Furthermore, *α*-synuclein aggregates in astrocytes contributing to such dysfunction [11].

This review aims at summarizing the evidence for astrocyte participation in experimental PD genesis, the probable neuroprotective effect of molecules like GDNF (glial-derived neurotrophic factor), MANF (mesencephalic astrocyte-derived neurotrophic factor), and CNTF (ciliary neurotrophic factor), and the involved pathological cascades described so far, illustrating the potential use of these findings in developing new-generation neuroprotective agents. Following PubMED searches performed using “Parkinson's Disease, astrocytes, molecular signaling” strings, relevant papers published in English or Spanish before January 1, 2018, were included, while reference sections were also scrutinized out of these publications for new studies.

## 2. Role of Astrocyte Dysfunction in the Genesis of Experimental Parkinson's Disease

The glia accounts for over 50% of brain cells, comprising various cell subtypes, of which astrocytes are the most abundant [12, 13]. Although astrocytes were documented 100 years ago, relatively few studies have been designed to dig into their role in neurological disorders and diseases over time. Astrocytes can be both helpful and harmful in PD [14, 15], and a key aspect of PD pathophysiology is neuroinflammation in the central nervous system (CNS), for long considered a downstream response to dopaminergic neuronal death, definitely including the concurrence of reactive astrocytes [16, 17]. However, ongoing evidence suggests that astrocytes have a role in setting up PD pathophysiology. Astrocytes may have neuroprotective effects by producing factors like the glial cell line-derived neurotrophic factor (GDNF) [18], the mesencephalic astrocyte-derived neurotrophic factor (MANF) [19], and the ciliary neurotrophic factor (CNTF) [20]. Recently, a relative increase in the astrocytic level of senescence markers, inflammatory cytokines, and metalloproteinases was observed on postmortem substantia nigra specimens of five PD patients compared with five controls, illustrating astrocytes' relevance in PD [21]. Furthermore, astrocytes and fibroblasts developed senescent phenotypes when exposed to the neurotoxin paraquat in human cell cultures, and conversely, neurodegeneration was attenuated in response to paraquat in a senescent astrocyte-selectively depleted mouse model [21].

This section reviews evidence from a molecular signaling perspective about the participation of astrocytes in the genesis of experimental PD and the involved molecular cascades.

### 2.1. Wnt/*β*-Catenin Signaling Cascade

The Wnt1 (wingless-type MMTV (mouse mammary tumor virus) integration site family, member 1) pathway has emerged as an essential signaling cascade regulating differentiation, neuron survival, axonal extension, synapse formation, neurogenesis, and many other processes in developing and adult tissues [22]. Little is known on the role of Wnt agonists in the midbrain [23]. In healthy human progenitor-derived astrocytes (PDAs), *β*-catenin leads to modulation of genes relevant to regulating aspects of glutamate neurotransmission [24]. However, the expression of Wnt components in adult astrocytes [25, 26] and the identification of activated midbrain astrocytes as candidate components of Wnt1 signaling suggest that astrocytes may be relevant sources of Wnt1 [27]. Using the proneurotoxin MPTP- (1-methyl-4-phenyl-1,2,3,6-tetrahydropyridine-) lesioned mouse model, 92 mRNA species molecular profiling in the midbrain revealed a specific, robust, and persistent increase in the expression of the canonical Wnt1 agonist, but not of Wnt3a or Wnt5a, during MPTP-induced dopaminergic degeneration [28]. The activated astrocytes rescued mesencephalic dopaminergic neurons from MPP+-induced tyrosine hydroxylase-positive (TH^+^) neuron toxicity promoting dopaminergic neurogenesis through Wnt1/*β*-catenin signaling activation [28]. Further evidence supports that the Wnt signaling system may be reinforced following injury in the adult CNS [29]. Likewise, some studies suggest that Wnt/*β*-catenin activation reduces neurodegeneration in mouse models of Alzheimer's disease [30, 31].

Growing evidence endorses the critical participation of Wnt1 in PD genesis. The neuroprotective effects of the Wnt pathway could be blocked by a Wnt1 antibody [28], and also, the Wnt1-targeted interfering RNA-induced Wnt1 depletion in midbrain astrocytes resulted in a substantial decrease in TH^+^ neuron survival upon serum deprivation and 6-OHDA or MPP+ treatment in neuron-astrocyte cocultures [32].

Furthermore, the Fzd-1 immunofluorescent signal largely increased in the rescued TH^+^ neurons in dopaminergic DA neurons cocultured with midbrain astrocytes, oppositely to the dramatic Fzd-1 receptor downregulation observed in purified neurons, either in vitro or in vivo, following the neurotoxic insult [32].

**Figure 1:**
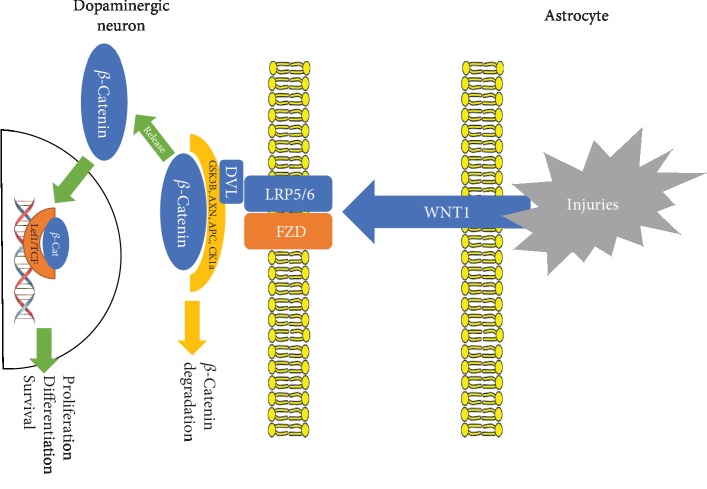
Wnt/*β*-catenin signaling cascade in Parkinson's disease. Upon insult, Wnt/*β*-catenin activation stimulates neurogenesis in mouse models of Parkinson's and Alzheimer's diseases. In mammalians, the signaling pathway is activated when the Wnt ligand binds to its Frizzled (FZD) receptor. The protein complex of FZD, LRP5/6, CK1, and GSK-3*β* marks *β*-catenin protein for degradation in the proteasome. Unless *β*-catenin undergoes degradation, it will be translocated to the nucleus to regulate the proliferation and survival of dopaminergic neurons.

Interestingly, exogenous activation of Wnt signaling with a specific GSK-3*β* (glycogen synthase kinase 3) antagonist sharply amplified astrocyte-induced DA neuroprotection in MPP+-treated astroglia-neuron cocultures. Glial inserts or Wnt1 direct addition to purified DA neurons just before MPP+ insult largely conferred neuroprotection, which was blocked by a Wnt1 antibody or the Wnt antagonist Fzd-1-cysteine-rich domain, supporting the critical role of Wnt1 in dopaminergic neuron survival [28]. Over and above, pharmacological inhibition of GSK-3*β* activity increased neuroblasts' population and promoted their migration towards the rostral migratory stream and the lesioned striatum in PD animal models [33]. Inhibiting GSK-3*β* enhanced dendritic arborization and survival of the granular neurons and stimulated neural stem cell-to-neuronal phenotype differentiation in the hippocampus of PD animal models.  Figure 1 summarily illustrates the Wnt/*β*-catenin/Fdz-1 pathway.

### 2.2. Transient Receptor Potential Vanilloid 1 (TRPV1)

**Figure 2:**
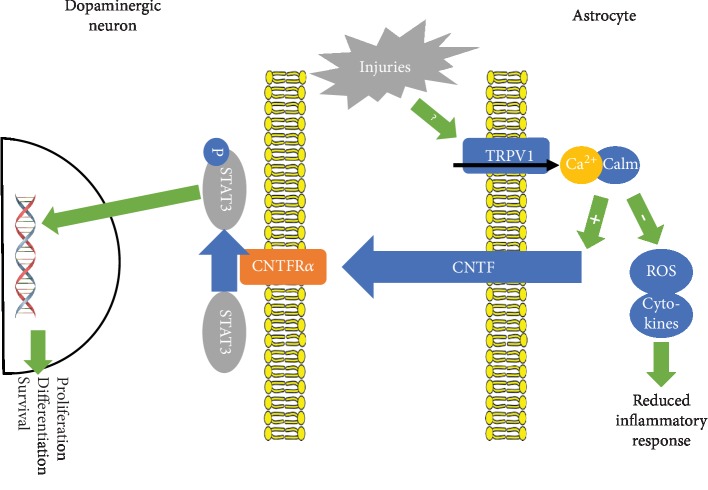
TRPV1-CNTF signaling cascade in PD. Capsaicin-mediated stimulation of TRPV1 through activation of CNTFR*α* and the STAT pathway increases dopaminergic neuron viability in PD rat models. Activation of TRPV1 has also been associated with a reduced expression of the proinflammatory cytokines and reactive oxygen species/reactive nitrogen species in a PD rat model. TRPV1: transient receptor potential vanilloid 1 channel; CNTFR*α*: ciliary neurotrophic factor receptor *α* subunit.

Transient receptor potential vanilloid 1 (TRPV1), the capsaicin receptor, is involved in nociception, is highly expressed in sensory neurons [34], and may also modulate neuronal function in other brain areas [35], control motor behavior [36], and regulate neuroinflammation [37]. The TRPV1 channel is expressed in neuronal and nonneuronal cells, where it is involved in the regulation of neurotransmitter release, and postsynaptically, where it influences neurotransmitter signaling [38]. In astrocytes, TRPV1 channels are responsible for Ca^2+^ entry from the extracellular space, accounting for nearly 20% of total Ca^2+^ events occurring in hippocampal astrocytes. Besides, the TRPV1 channels have been linked to some forms of long-term potentiation of glutamatergic transmission and GABAergic transmission regulation [39, 40]. Capsaicin-mediated activation of TRPV1 on astrocytes increases CNTF endogenous synthesis in vivo, increasing dopaminergic neuron viability through activation of the CNTF receptor alpha subunit (CNTFR*α*) and preventing neurodegeneration after MPP+ and 6-OHDA administration in PD rat models [41, 42]. Activation of TRPV1 in a PD rat model was recently associated with a reduced expression of the TNF-*α* and interleukin-1*β* proinflammatory cytokines, the reactive oxygen species/reactive nitrogen species (ROS/RNS) generated by NADPH oxidase at the microglia, and the inducible nitric oxide synthase or reactive astrocyte-derived myeloid peroxidase [43]. The relevance of this pathway to PD is further supported by the increased TRPV1 and CNTF levels in GFAP^+^ (glial fibrillary acidic protein-positive) astrocytes and CNTFR*α* on dopaminergic neurons found in PD patients [41]. The TRPV1-CNTF pathway is summarized in Figure 2.

### 2.3. The JWA Gene (ADP-Ribosylation-Like Factor 6 Interacting Protein 5)

Oxidative damage has been considered a primary pathogenic mechanism of nigral dopaminergic neuronal cell death in PD [44]. At the molecular level, both DNA damage and abnormal activation of the known mediator of tissue damage and inflammation NF-*κ*B (nuclear factor kappa-light-chain-enhancer of activated B cells) have been implicated in oxidative damage [45]. The NF-*κ*B protein complex exists as a cytoplasmic p50/p65 heterodimer which binds to the I*κ*B inhibitory subunit [46]. The activation of NF-*κ*B is mediated by the upstream I*κ*B kinase (IKK), a heterotrimer made of 2 catalytic subunits, IKK*α* and IKK*β*, and the NF-*κ*B essential modulator regulatory IKK*γ* subunit [47]. Exposure to various stimuli like oxidative stress, proinflammatory cytokines, and growth factors induces IKK phosphorylation, leading to I*κ*B polyubiquitination and proteasomal degradation. In turn, I*κ*B degradation induces NF-*κ*B translocation to the nucleus, where NF-*κ*B binds to its cognate DNA sequences and its coactivators to ultimately regulate gene expression [48].

**Figure 3:**
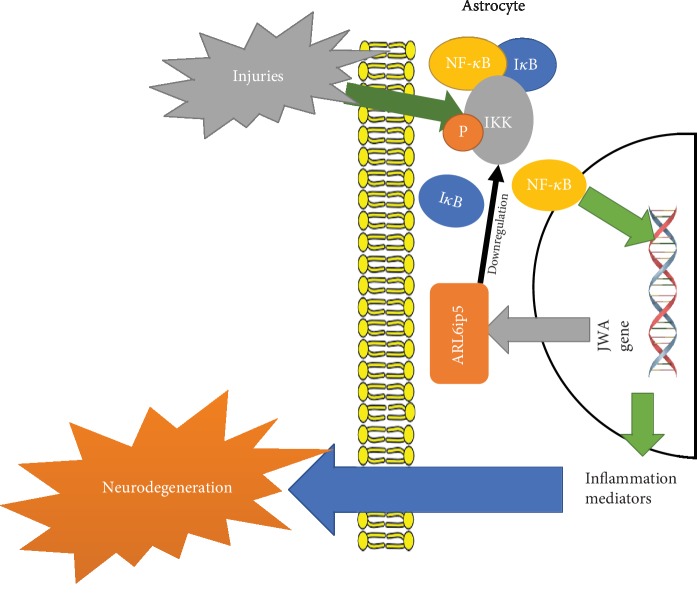
NF-*κ*B/JWA/ARL6ip5 signaling pathway in PD. Both DNA damage and abnormal activation of the known mediator of tissue damage and inflammation NF-*κ*B have been implicated in oxidative damage. The ARL6ip5 downregulates IKK*β* expression inhibiting NF-*κ*B signaling pathway activation. NF-*κ*B: nuclear factor kappa-light-chain-enhancer of activated B cells; ARL6ip5: ADP-ribosylation-like factor 6 interacting protein 5.

The ARL6ip5 (ADP-ribosylation-like factor 6 interacting protein 5) or JWA gene codes for a novel microtubule binding protein regulating cancer cell migration via MAPK cascades [49] and mediating leukemic cell differentiation [50, 51]. It is also a key regulator of base excision repair of oxidative stress-induced DNA damage by XRCC1 (X-ray repair cross-complementing 1) stability regulation [52, 53]. Miao and colleagues reported that JWA knockout (KO) astrocytes showed NF-*κ*B pathway activation in dopaminergic neurons and neurodegeneration [54], suggesting JWA downregulation of the NF-*κ*B signaling pathway [54]. Indeed, JWA downregulated the expression of IKK*β* inhibiting NF-*κ*B signaling pathway activation [54].  Figure 3 summarizes the JWA/NF-*κ*B pathway.

### 2.4. Nrf2-ARE Pathway in Parkinson's Disease

**Figure 4:**
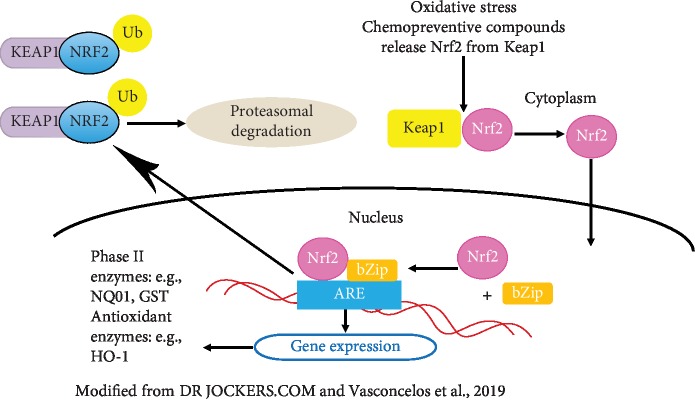
The Nrf2-ARE signaling pathway in Parkinson's disease. Under unstressed conditions, oxidative molecules like ROS and RNS activate the protective antioxidant pathway, dissociating the cytosolic Nrf2/Keap1 complex. The Nrf2 factor translocates to the nucleus where associated with bZip proteins trigger the expression of several homeostatic genes with the ARE sequence in their promoters, including SOD, HO-1, GST, and NQO1. Upon inactivation, Nrf2 is sequestered by Keap1 and targeted for ubiquitination and proteasomal degradation. Nrf2: nuclear factor (erythroid-derived 2)-related factor 2; Keap1: Kelch-like ECH-associated protein 1; bZip: basic region leucine zipper (bZip) transcription factors; SOD: superoxide dismutase; HO-1: heme oxygenase-1; GST: glutathione S-transferase; NQO1: NAD(P)H: quinone oxidoreductase-1.

Free radicals, regularly produced at physiological levels, are required for signaling and plasticity in the healthy brain. However, oxidative stress appears when their production exceeds the cellular antioxidant defense. High levels of free radicals are neurotoxic leading to pathological processes and cell death in time. Oxidative stress has been associated with neuronal death and involved in the pathogenesis of multiple chronic neurodegenerative diseases including Alzheimer's disease, PD, Huntington's disease, amyotrophic lateral sclerosis, and neurological illnesses [55, 56]. The nuclear factor erythroid 2-related factor 2 (Nrf2) antioxidant response element (ARE) is a key in the Nrf2 antioxidant system pathway upregulating an array of antioxidant and detoxifying enzymes. Currently, as Nrf2 is considered a possible therapeutic target for treating oxidative stress-related disorders, some studies have targeted Nrf2 to confer neuroprotection in PD [55, 56]. The Nrf2 factor counteracted PD-related neuronal cell death through the expression of cytoprotective genes with anti-inflammatory and antioxidant properties. Data from postmortem PD human brains and Nrf2 knockout mice indicate an association between Nrf2-ARE pathway dysfunction and PD pathogenesis [57]. An Nrf2 deficiency increases MPTP sensitivity and exacerbates vulnerability to 6-OHDA both in vitro and in vivo. Transplants of astrocytes overexpressing Nrf2 were protected from 6-OHDA-induced damage in the living mouse [58–61]. In postmortem brains of PD patients, p62 (nucleoporin p62) and NQO1 (NAD(P)H dehydrogenase [quinone] 1) were found partially sequestered in Lewy bodies, indicating that Nrf2 compromised neuroprotective capacity [62]. Also, Nrf2 activation by dimethyl fumarate protected the substantia nigra neurons against *α*-synuclein toxicity in a murine PD model, an effect not evident in Nrf2-knockout mice [62, 63]. The activation of Nrf2 upregulated brain heme oxygenase-1 (HO-1) and NQO1 and prevented MPTP-induced neuronal death in the substantia nigra [62, 63]. Likewise, Nrf2-ARE pathway activation by siRNA (small double-stranded interfering RNAs) knockdown of Keap1 (Kelch-like ECH-associated protein 1) reduced oxidative stress partially protecting from MPTP neurotoxicity [64]. Some studies suggest that Nrf2 activation in glial cells may be required to exert its protective effects in PD and PD models [60, 61]. However, glial Nrf2 nuclear translocation in the substantia nigra was not found in PD brains [65], and in vitro studies show that neuronal Nrf2 activation, even in the absence of glia, induces neuroprotection against oxidative damage triggered by parkinsonism-inducing neurotoxins [61, 66–70]. Uric acid activated the Nrf2-ARE pathway by increasing mRNA and the expression of Nrf2 and three Nrf2-responsive genes and inhibited oxidative stress in MPTP-treated mice improving behavioral performance and cognition. It also increased TH^+^ dopaminergic neurons and decreased GFAP^+^ astrocytes in the substantia nigra [71]. Astrocyte contribution to neuroprotection and the underlying neuroprotective mechanisms are yet to be studied. Glutathione secretion from astrocytes was increased following Nrf2-ARE activation in vitro [55].  Figure 4 summarizes the Nrf2-ARE pathway.

### 2.5. Other Pathways Associated with Astrocyte Dysfunction in PD

This section briefly reviews other less convincingly supported pathways as to their involvement in PD-related astrocytic dysfunction.

The toxic dopamine quinones resulting from cytosolic mismanagement of dopamine excess can perpetuate dopaminergic dysfunction in PD [72]. They can be competitively antagonized by other cysteine-rich molecules including superoxide dismutase, glutathione, and metallothioneins (MTs). They are a family of ubiquitous low-weight proteins, of which MT1 appears to be expressed in astrocytes in response to mechanical or toxic neuronal injury [43, 72, 73]. Interestingly, MT1 attenuated neurotoxin-induced neuronal death both in vivo and in vitro [72–74].

In humans, the deglycase DJ-1 protein is encoded by the PARK7 gene, whose mutation causes one of the hereditary forms of PD [75]. Interestingly, its overexpression in reactive astrocytes has been reported in sporadic cases of PD [75], suggesting a pathophysiological role in PD. Indeed, DJ-1 overexpression reduced rotenone neurotoxicity in neuron-astrocyte cocultures, whereas the opposite was found after DJ-1 deletion [75, 76]. Its exact mechanism of action in astrocytes remains elusive, some data suggesting an effect on mitochondrial function that in turn might favor the release of paracrine-acting molecules [76, 77].

The enzymatic protein thrombin plays a key role in the coagulation cascade and is upregulated upon CNS damage [78, 79]. In normal conditions, thrombin activates the protease-activated receptor (PAR) subtypes PAR-1, PAR-3, and PAR-4 [78, 79], although it may bind to PAR-2 at high concentration [79]. To date, the four known PAR subtypes are associated with G proteins and determine multiple cellular responses [78, 79].

Ishida and colleagues studied the presence of the thrombin-PAR pathway in human samples of the substantia nigra pars compacta [78]. The thrombin precursor prothrombin and the PAR-1 were observed only in astrocytes, expressed at a higher level for the latter along with a higher density of thrombin-positive vessels in PD brain specimens compared with controls. In astrocyte cultures, PAR-1 activation by thrombin increased GDNF and glutathione peroxidase expression, albeit not inflammatory molecules like IL-1b, IL-6, IL-8, and MCP-1 and the nerve growth factor level.

There is conflicting evidence on the neuroprotective potential of GDNF from astrocytic origin [80]. Pretreatment with GDNF attenuated neuronal death in dopamine-depleted corpus striatum [80–82] while the GDNF level in brain tissue from PD patients was comparable to that found in control patients and higher in the nigrostriatal dopaminergic region [83].

## 3. Restoring Astrocyte Function as a Preventive Strategy against Dopaminergic Neurodegeneration in Parkinson's Disease

As hereinabove discussed, astrocytic dysfunction may largely contribute to dopaminergic neurodegeneration [10, 16, 17]. The astrocytes release Wnt1 which may lengthen dopaminergic neuron survival by activating Fzd-1 receptors [84]. Addition of Wnt1 to purified DA neurons prevented MPP+ neurotoxicity [28], likely disclosing a promising neuroprotective therapy in PD and warranting clinical studies which, at present, are lacking in this regard.

Capsaicin-mediated activation of astrocytic TRPV1 is followed by CNTF release and CNTFR*α* activation on dopaminergic neurons whose viability increases [41]. Indeed, pretreatment with capsaicin 0.5 mg/kg largely reduced dopaminergic neurons' death and improved behavioral outcomes in MPTP-lesioned mice [43], while treatment with TRPV1 antagonists capsazepine and iodine-resiniferatoxin reversed both effects. Similar results were observed in 6-OHDA-lesioned mice [41, 42]. Capsaicin increased superoxide dismutase and catalase levels and decreased lipid peroxidation in the brain, suggesting an antioxidant effect [42].

Knocking down JWA in astrocytes has also been related to DA neurodegeneration, likely by NF-*κ*B disinhibition [85]. Provided that NF-*κ*B is a potent proinflammatory molecule [85], even though neuroprotection following exogenous JWA or related compounds administration has not been reported, a new experimental PD model unrelated to dopaminergic neurotoxins may stem out of the above.

Large evidence supporting astrocyte involvement in the genesis of experimental PD comes from cell culture studies sparing any interaction with the glia which is functional in the brain.

The potential neuroprotective effect of GDNF was studied by inducing its expression in astrocytes through vector transfection in 6-OHDA- and MPTP-treated rats and mice, respectively [80]. Overexpression of GDNF prevented neurotoxicity, namely, neuronal death and behavioral abnormalities, even up to 14 weeks after transfection when astrocytic activation and astrogliosis were observed in the MPTP model [80].

Silibinin or silybin is the major active constituent of the standardized extract of the milk thistle seeds known as silymarin with potential hepatoprotective and antineoplasic effects [86, 87] which showed neuroprotective effects in MPTP-treated mice [88]. Silibinin also reduced glial activation, dependent on extracellular signal-regulated kinase (ERK) and c-Jun N-terminal kinase (JNK) stress response kinase activation [89, 90]. Accordingly, in vitro studies demonstrated that silibinin suppressed astroglial activation inhibiting ERK and JNK phosphorylation in primary astrocytes following MPP+ treatment [88].

Loss of Nrf2-mediated transcription exacerbated vulnerability to the neurotoxin 6-hydroxydopamine (6-OHDA) in a Parkinson mice model and N27 rat dopaminergic neuronal cell line. Also, astrocytes overexpressing Nrf2 transplantation induced the Nrf2-ARE pathway protecting from 6-OHDA-induced damage in the living mouse [61]. On the other hand, Keap1 siRNA administration in striatum primary astrocytes upregulated the Nrf2-ARE pathway, protected from oxidative stress, and modestly spared from MPTP-induced dopaminergic terminal damage [64]. Uric acid also exerted a neuroprotective effect improving behavior and cognition in MPTP mice, increased TH^+^ dopaminergic neurons, and decreased GFAP^+^ astrocytes in the substantia nigra [71]. All in all, experimental evidence supports a key role for astrocytes in the Nrf2-ARE and neuroprotection. The Nrf2-ARE pathway poses as a promising therapeutic target for reducing or preventing cell death in PD.

Table 1 summarizes astrocyte-interacting drugs with possible neuroprotective effects.

## 4. Conclusion

Despite the fact that astrocytes, the most abundant glial cells, are critical for brain function, their role in PD was long considered a byproduct of neuroinflammation. However, the bulk of ongoing evidence suggests that astrocyte dysfunction might occupy a central position in the genesis of experimental PD [14, 15].

Three main pathways contributing to PD development involving astrocytes could be identified. Firstly, noxious stimuli increase Wnt1 synthesis in astrocytes [27, 28] modifying gene expression in DA neurons upon Fzd receptor activation and *β*-catenin nuclear translocation [32]. Secondly, noxious stimuli and perhaps inflammation too stimulate astrocytic TRPV1 reducing oxidative species generation, releasing CNTF [41, 42], modifying gene expression, and improving dopaminergic neuron survival and viability [41]. Last but not least, the JWA gene induces astrocytic ARL6ip5 synthesis, which inhibits IKK*β* lowering the level of the active NF-*κ*B level [54], a potent inductor of inflammatory responses. The relevance of other pathways involving metallothioneins, DJ-1 protein, thrombin, and GDNF is less clear, though might turn out as equally important.

The pursuit of neuroprotective strategies in PD is a top priority as once and again negative results have been obtained so far [91]. The pathways herein discussed disclose interesting targets to be explored in this regard. Certain molecules like capsaicin [43] and silibinin [88] have shown unquestionably interesting effects in rodent PD models. They are naturally found in chili peppers and cardum, respectively; they have sometimes been used for therapeutic purposes. Needless to say that before clinical trials in PD may be envisaged, studies in primate PD models are needed. Results are hitherto encouraging, and more data are hopefully coming forth in the near future. Overexpression of GDNF by vector transfection has also shown some efficacy in rodent models [80] contrasting with the lack of clinical benefit after intraputaminal or intracerebroventricular infusions of GDNF in PD patients [92, 93]. Nevertheless, an eventual benefit from GDNF infusion might be limited by its reach to and bioavailability at the site of interest, making drug delivery a crucial aspect of GDNF therapy worth exploring.

Knocking out JWA increased NF-*κ*B activity in DA neurons [54] presumably depicting a new PD model, eventually surpassing the limitations of neurotoxin PD models which do not accurately reproduce full PD pathophysiology [94]. The JWA knockout mouse developed a PD-like phenotype with selective loss of dopaminergic neurons in the substantia nigra pars compacta and monoaminergic neurotransmitter level in the corpus striatum [85]. Constitutive expression of NF-*κ*B, a known promoter of inflammatory responses, participates in neurogenesis, neuritogenesis, and plasticity while inducible NF-*κ*B expression leads to glial proinflammatory responses, neuronal proapoptotic responses and death, vascular inflammation, and increased endothelial permeability [95]. Inducing experimental inflammation, a PD hallmark [96], might advantageously reproduce the whole spectrum of the disease bearing other brain areas compromised. Further research is warranted to fully characterize this plausible new model.

## Figures and Tables

**Table 1:** Potentially neuroprotective molecules upon astrocytic behavior modification.

Molecule	Proposed mechanism of action	Tested PD models
Capsaicin	Activation of TRPV1 in astrocytes	MPTP (mouse), 6-OHDA (rat)
GDNF (vector transfection)	GDNF overexpression in astrocytes	MPTP (mouse), 6-OHDA (rat)
Silibinin	Suppression of astrocyte activation (via ERK/JNK phosphorylation)	MPTP (mouse)

GDNF: glial cell line-derived neurotrophic factor; MPTP: 1-methyl-4-phenyl-1,2,3,6-tetrahydropyridine; TRPV1: transient receptor potential vanilloid 1 channel; ERK/JNK: extracellular signal-regulated kinase/c-Jun N-terminal kinase.
